# Development of a mammalian cell culture process for rapid Clinical-Scale production of novel Influenza Nanoparticle vaccines

**DOI:** 10.1186/1753-6561-9-S9-O12

**Published:** 2015-12-14

**Authors:** Payal Biswas, Christian Trozado, James Lee, Richard M Schwartz

**Affiliations:** 1 Vaccine Production Program, Vaccine Research Center, NIAID, NIHb9 West Watkins Mill Road, Suite 250, Gaithersburg, MD-20878, USA

## Background

Influenza virus infections cause seasonal epidemics, affecting millions of people worldwide. The World Health Organization (WHO) estimates ∼300,000-500,000 deaths per year worldwide due to seasonal influenza and more than $26.8-87.1 billion/year in healthcare costs in the United States alone [1]. Influenza, a segmented RNA virus achieves part of its ongoing virulence as a result of its strikingly high mutation rate, typically reported for Influenza A viruses of ~2 to 2.5 × 10−3mutations/site/year. Thus, is the intense effort and energy devoted to achieving effective long-term human and veterinarian vaccines. Currently, licensed influenza vaccines are either trivalent (three influenza strains) or quadrivalent (four influenza strains), as either an injectable inactivated whole virus (IIV) or a nasal spray live attenuated vaccine (LAIV) [2]. Although efficacious, standard traditional Influenza vaccine production is laborious, only moderately high-throughput and requires physical plants housing and/or incubating millions of specific-pathogen-free eggs. Research targeting alternate strategies has been prodigious, both at the preclinical and even Phase I clinical level, and has investigated approaches such as split virion, subunit, DNA, and viral vectored vaccines. Among the recently-explored, more novel and potentially-promising strategies has been recombinant particulate vaccines generally comprising virus-like particles or nanoparticles.

At the Vaccine Research Center (VRC), National Institute of Allergy and Infectious Disease (NIAID), National Institutes of Health (NIH), fusion proteins of N-terminal Influenza Hemagglutinin (HA) residues and C terminal H. pylori ferritin residues have been conceived, designed, expressed and determined to assemble into ferritin-like cage nanoparticles termed hemagglutinin ferritin nanoparticles (HAF-NP). HAF-NP used as immunogens in mouse immunization studies, elicited antibody titers more than seven-fold higher and neutralizing antibody titers with both extended breadth and greater potency than the seasonal trivalent inactivated vaccine. Neutralization of H1N1 viruses extended to viruses from 1934 to 2007, and ferrets were protected by HAF-NP vaccination against a heterologous virus [3].

## Experimental approach

To enable HAF-NP to proceed to Phase I clinical trials, the Vaccine Production Program (VPP), VRC has developed manufacturing processes amenable to GMP manufacture and FDA clearance for human clinical trials. Among those processes, a transient gene expression (TGE) system based on Polyethylenimine (PEI)-mediated transfection for cell culture-based production of HAF nanoparticles was developed. TGE was selected both because of its short production time compared to stable cell line generation and because of the specific requirements of vaccine manufacture. In rodent cell lines such as Chinese Hamster Ovary, FDA requires that viral inactivation or clearance of endogenous rodent viruses be demonstrated, which may potentially compromise the structural integrity of such an oligomeric 24-gon. To address this problem for production, the human cell line HEK293, harboring only the retroviruses endogenous to those present in the clinical subjects, is commonly used as a host cell. In addition, HEK293 cells have been shown to have higher transfection efficiencies and are therefore typically used for TGE production. Thus, HEK293-based TGE was selected as the method of choice to produce HAF. The different HA-F constructs used were- H1 A/California/04/09, H2A/Singapore/1/57, H1 A/New Caledonia/20/99 and H5 A/Indonesia/05/05.

## Results and conclusions

Transfection conditions, media, feed, process parameters and scale up conditions were optimized to achieve a high titer process. The effect of DNA to PEI ratio on nanoparticle production was evaluated and the most optimal DNA to PEI ratio was determined as 1:2 for HAF nanoparticle production. In addition, a pDNA:PEI ratio of 20 mg/L:40 mg/L was optimized for the transient transfection process. For the production medium and feed, chemically-defined supplements were explored, avoiding both animal-origin reagents and the subsequent FDA requirement to demonstrate their elimination in the final drug substance and the lot-to-lot variability sometimes present in chemically-complex, undefined media and supplements. Data indicated that the chemically-defined supplement Cell Boost 5 (CB 5 from Hyclone) added to the basal production medium as well as in the production feed significantly increased the productivity of nanoparticle production. Process parameters and wave bag expression conditions were optimized for a consistent process. 50L-scale production of an influenza nanoparticle vaccine has been successfully completed for Phase I clinical trials.

To summarize, a mammalian cell culture based process was developed to ensure rapid, reliable and efficient production of novel influenza nanoparticle vaccines. A transient transfection platform process was optimized and scaled up for 50L scale production of different HA-F nanoparticles. Phase 1 clinical material has been successfully produced and is in pre-clinical studies.

**Figure 1:**
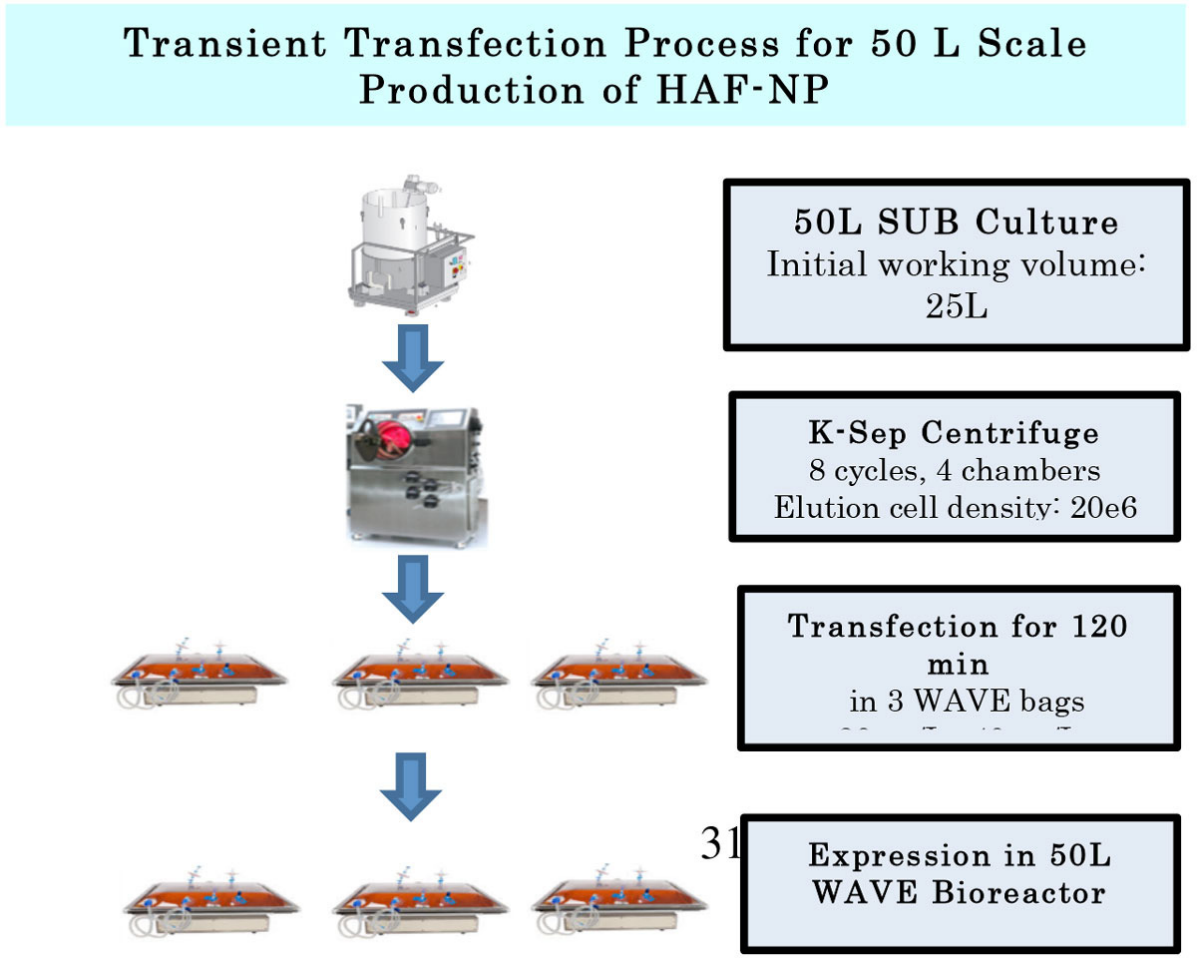
**A schematic representation of the transient transfection process is shown**. The HEK 293 cells were grown in a 50L Single Use Bioreactor (SUB). Cells were then washed and concentrated using a semi continuous centrifuge (kSep) and harvested into 3 Wave bags. Transfection was carried out in the 3 Wave bags by adding pDNA and PEI. After 2 hours of incubation, production media was added. To facilitate expression, Valproic acid (VPA) and production feed were added at 24 and 48 hours post-transfection, respectively and culture was harvested at 96 hours post-transfection.

## References

[B1] Molinari NA Ortega-Sanchez IR Messonnier ML Thompson WW Wortley PM Weintraub E Bridges CB The annual impact of seasonal influenza in the US: measuring disease burden and costs Vaccine 2007 25 5086 5096 17544181 10.1016/j.vaccine.2007.03.046

[B2] Sanjuán R1 Nebot MR Chirico N Mansky LM Belshaw R Viral mutation rates J Virol 2010 84 9733 9748 20660197 10.1128/JVI.00694-10 PMC2937809

[B3] Kanekiyo M Wei CJ Yassine HM McTamney PM Boyington JC Whittle JR Rao SS Kong WP Wang L Nabel GJ Self-assembling influenza nanoparticle vaccines elicit broadly neutralizing H1N1 antibodies Nature 2013 499 102 106 23698367 10.1038/nature12202 PMC8312026

